# Quantifying TERT promoter mutations in tumor-derived DNA shed into the oral cavity as a potential biomarker for oral squamous cell carcinoma

**DOI:** 10.3389/fonc.2025.1720783

**Published:** 2025-12-19

**Authors:** Noemy Starita, Marta Tagliabue, Tarik Gheit, Andrea Cerasuolo, Sara Amiranda, Tiziana Pecchillo Cimmino, Luisa Dassi, Anna Lucia Tornesello, Rita De Berardinis, Fausto Maffini, Giuseppe De Palma, Stefania Vecchio, Angelo Paradiso, Giovanni Blandino, Massimo Tommasino, Mohssen Ansarin, Susanna Chiocca, Maria Lina Tornesello

**Affiliations:** 1 Molecular Biology and Viral Oncology Unit, Istituto Nazionale Tumori IRCCS Fondazione G. Pascale, Napoli, Italy; 2 Division of Otolaryngology and Head and Neck Surgery, European Institute of Oncology IRCCS, Milano, Italy; 3 Epigenomics and Mechanisms Branch, International Agency for Research on Cancer (IARC), Lyon, France; 4 Innovative Immunological Models Unit, Istituto Nazionale Tumori IRCCS Fondazione G. Pascale, Napoli, Italy; 5 Department of Surgical Pathology, European Institute of Oncology IRCCS, Milan, Italy; 6 Institutional BioBank, Experimental Oncology and Biobank Management Unit, IRCCS Istituto Tumori “Giovanni Paolo II”, Bari, Italy; 7 Medical Oncolgy Unit, IRCCS Ospedale Policlinico San Martino, Genoa, Italy; 8 Translational Oncology Research Unit, IRCCS, Regina Elena National Cancer Institute, Rome, Italy; 9 Viruses and Cancer Unit, Department of Experimental Oncology, European Institute of Oncology IRCCS, Milano, Italy

**Keywords:** head and neck squamous cell carcinoma (HNSCC), oral squamous cell carcinoma (OSCC), oropharyngeal squamous cell carcinoma (OPSCC), TERT promoter mutations, prognostic biomarkers

## Abstract

**Background:**

Head and neck squamous cell carcinomas (HNSCC) have high recurrence and poor prognosis, largely due to delayed diagnosis. Identification of somatic mutations and human papillomavirus (HPV) sequences in tumor DNA shed in the oral cavity may provide non-invasive biomarkers for early HNSCC detection.

**Objectives:**

The study aimed to evaluate TERT promoter (TERTp) mutations in tumor DNA extracted from oral rinses as potential biomarkers for head and neck cancers.

**Methods:**

TERTp mutations (C228T and C250T) were examined in DNA extracted from oral rinses of 132 HNSCC patients, of whom 63 had paired tumor tissue available for analysis, and from four head and neck squamous cell carcinoma derived cells lines (CAL27, SCC152, SCC154, FaDu) by using droplet digital PCR (ddPCR). TERT gene expression was analyzed in all cell lines by real time PCR. Associations with tumor site, smoking status, and sex were evaluated, and mutant allele frequencies (MAF) quantified.

**Results:**

TERTp mutations were identified in 25% of oral rinses (33 out of 132, 95%CI 22.7 - 46.3) and in 27% of tumor tissues (17 out of 63, 95%CI 9.9 - 27.2). Mutation rates were highest in oral SCC (OSCC), present in 50% of oral rinses (n=25/50, 95%CI 16.2 - 36.9) and 46% of matched tumor tissues (n=13/28, 95%CI 6.9 - 22.2), with 96% concordance (kappa value 0.86, 95%CI 67-100). MAF were higher in tumor tissues and correlated with levels in corresponding oral fluids. Mutations were uncommon in non-OSCC cases, being detected in 9.7% of oral rinses and 11% of tumor tissues. In OSCC, TERTp mutations were more frequent in males. The CAL27 cell line carried the TERTp C228T mutation and TERT mRNA expression was 11–15 folds higher compared to non-mutated oral carcinoma cell lines.

**Conclusions:**

TERTp C228T and C250T are mutually exclusive and occur at a high frequency in oral rinses and tumor tissues of OSCC patients, showing high concordance between paired samples. These findings support the potential of TERTp mutations as non-invasive biomarkers for OSCC detection. Moreover, their higher prevalence in males suggests possible sex-related differences in OSCC mutation patterns.

## Introduction

Head and neck cancers are the sixth most common type of tumor worldwide, with over 800,000 new cases and a mortality rate exceeding 450,000 in 2022 (1). The majority of head and neck tumors are squamous cell carcinoma (HNSCC) originating from the mucosa of the lip and oral cavity (389,485 cases), larynx (n= 188,960), nasopharynx (n= 120,416) and oropharynx (n= 106,316) (1). The incidence of HNSCC at each site varies in diverse populations, mainly reflecting differences in established risk factors (2). Tobacco and alcohol consumption as well as human papillomavirus (HPV) infection are the primary causes of oral SCC (OSCC) and oropharyngeal SCC (OPSCC), respectively (3). Both OSCC and OPSCC have higher rates in North America and Europe, and HPV-positive OPSCC are more common in younger patients in developed countries (4). Detection of HPV in body fluids has been demonstrated to be effective as minimally invasive diagnostic tool for detecting HPV-related OPSCC (5). However, no validated screening protocols have yet been implemented for the non-invasive diagnosis of the most common type of HNSCC, namely oral squamous cell carcinoma.

Mutation profiling of circulating tumor DNA is being used as a promising diagnostic and screening strategy in many cancer types (6). Novel techniques enabling high-throughput detection of small quantities of nucleic acids shed from cancer tissues into fluid matrices could serve as a promising tool for early tumor diagnosis (6). Methods based on the analysis of circulating tumor DNA have the potential to significantly improve cancer screening, particularly for those cancers such as HNSCC that currently do not have screening approaches (7). Diagnosing cancers at early stages based on their molecular signatures detected in biological fluids, offers a unique opportunity to improve survival rates and reduce treatment-related morbidity (8).

Telomerase activity is upregulated in many tumor types, including HNSCC, through various mechanisms including chromosomal rearrangements, amplification of TERT gene, and activating mutations in the regulatory region of TERT gene (6–8). In addition, in HPV-related tumors the viral oncoprotein E6 has been shown to de-repress the TERT promoter (TERTp) and to re-activate TERT gene expression in epithelial cells through its interaction with MYC and NFX-1 thus contributing to cell immortalization (9). Recurrent activating mutations in TERTp, first identified in melanoma by Horn et al. (2013) and Huang et al. (2013), are among the most prevalent genetic alterations across various cancers, including OSCC (10–13). These mutations lead to the permanent activation of telomerase expression, by creating *de novo* consensus binding sites for the E-twenty-six transcription factors (ETS), resulting in proliferative cell immortality of somatic cells (14–16). Moreover, cell lines with TERTp mutations exhibit unique gene and protein expression profiles, which likely influence their biological characteristics (17). TERTp mutations have been frequently identified in OSCC (18, 19). However, the potential of detecting TERTp mutations in tumor DNA shed into oral fluids as biomarkers for OSCC detection remains mostly unexplored.

We previously demonstrated that frequency of TERTp mutations was significantly higher in OSCC compared to non-OSCC cases (18). In this study, we further analyze the distribution of TERTp mutations in oral, hypopharyngeal, laryngeal, and oropharyngeal SCC tissues and derived cell lines. In addition, we have analyzed the presence of TERTp mutations in tumor DNA extracted from oral rinses of HNSCC patients. Then, we have evaluated the concordance of TERT promoter mutations in DNA extracted from matched oral rinse and tumor tissue samples of HNSCC patients. Such analysis is essential to establish the role of TERTp mutations as potential biomarkers for early detection of head and neck cancers.

## Material and methods

### Study design, patients and cell lines

The study included 141 participants, of whom 132 patients were diagnosed with HNSCC comprising oral cavity SCC (OSCC, n=50), oropharyngeal (n=24, OPSCC) and oro-hypopharyngeal (n=1, OHSCC) SCC collectively named OPSCC (n=25), hypopharyngeal SCC (HPSCC, n=10), laryngeal SCC (LSCC, n=45) and unknown site HNSCC (n=2). The remaining 9 participants were diagnosed with hyperplasia or dysplasia (n=5), lymphoma (n=1) or normal tissue (n=3), and were collectively categorized as non-HNSCC. The patients were consecutively enrolled between 2019 and 2022 at the Division of Otolaryngology and Head and Neck Surgery of the IRCCS European Institute of Oncology (IEO), Milan, Italy. Criteria to be included in the study were age ≥ 18 years, have a suspected head and neck neoplasia, have not received any prior treatment for head and neck cancer. HNSCC diagnosis was determined on the basis of clinical, radiological, and cyto-histological examinations. All cases were staged clinically as previously reported (5). Oral rinse specimens were collected from 141 participants and matched formalin-fixed and paraffin-embedded (FFPE) cancer tissues were available for a sub-group of 63 patients. The study was approved by the IEO Ethical Committee (code IEO 1572). Participants signed an informed consent and gave permission to use the data and biological samples collected during the study. The study was performed in accordance with the Declaration of Helsinki. All oral fluids and FFPE tissues were previously analyzed for HPV DNA and results reported by Galati et al. (5).

The human squamous cell carcinoma cell lines SCC152 (ATCC^®^ CRL- CRL-3240^™^), SCC154 (ATCC^®^ CRL-3241™), CAL 27 (ATCC^®^ CRL-2095^™^) and FaDu (ATCC^®^ HTB-43^™^) were cultivated in Dulbecco’s modified Eagle’s medium containing 4.5g/L glucose, 10% fetal bovine serum, 100U/mL penicillin-streptomycin and cultured under standard conditions (pH 7.4, 37°C, 5% CO2).

### Oral sample collection and DNA extraction

Participants provided oral specimens by gargling 15 mL of 0.9% NaCl sterile solution in the oral cavity for 15 seconds. Then, the oral fluids were processed according to previously established protocols (5). Genomic DNA was extracted from oral fluids using the EZ1 DNA Tissue Kit and the BioRobot EZ1 (Qiagen, Hilden, Germany) according to the manufacturer’s recommendations. Briefly, samples were centrifuged at 6,000 rpm for 10 minutes, pellets were resuspended in the buffer G2 (Qiagen, Hilden, Germany) containing proteinase K and incubated at 56 °C for 3 hours (20). Purified DNA was eluted into 50μL of elution buffer.

### Tumor tissue collection and DNA extraction

A total of 63 available FFPE tumor tissue blocks have been sectioned, as previously described (21). From each paraffin block, a single initial 5 µm section, four consecutive 10 µm sections, and a final 5 µm section were sequentially cut. The initial and terminal sections were stained with hematoxylin and eosin and evaluated by pathologists, showing neoplastic tissue content above 70%, while the intermediate sections were collected for DNA extraction. Briefly, tissue sections and 5x10^6 cultured cells were incubated with proteinase K (0.2mg/ml at 37 °C overnight) in 200 µl of lysis buffer (50 mM Tris-HCl pH 8.5, 1 mM EDTA, 0.5% Tween 20) (22). DNA was extracted from each digested sample by phenol-chloroform-isoamyl alcohol solution (25:24:1) followed by precipitation with 0.3 M sodium acetate (pH 4.6) in 90% ethanol. Total RNA was isolated from cultured cells using the RNeasy Mini Kit (Qiagen, Hilden, Germany). DNA and RNA purity as well as concentrations were determined spectrophotometrically with a NanoDrop 2000C instrument (Thermo Fisher Scientific, Waltham, MA, USA).

### TERTp mutation analysis by droplet digital PCR

The droplet digital PCR (ddPCR) reactions were performed by using the QX200 droplet digital PCR system according to the manufacturer instructions (Bio-Rad Laboratories, Hercules, CA, USA). TERTp mutations C228T and C250T were detected by using the dHsaEXD72405942 and dHsaEXD46675715 assays, respectively (Bio-Rad Laboratories, Hercules, CA, USA). Each ddPCR reaction has been carried out in 20μl volume containing 10μl of 2x ddPCR Supermix for Probes (No dUTP), 1μl of 20x mutant (FAM) and wild-type (HEX) primers, 2μl of 5M Betaine solution (Sigma Aldrich), 0.25μl 80mM EDTA, between 10 and 100 ng of DNA template and deionized distilled water. Each reaction mix was entirely transferred into a well of Droplet Generator Cartridge (Bio-Rad Laboratories, Hercules, CA, USA) and overlaid with 70μl of droplet generation oil for probes (Bio-Rad). The reaction mix was partitioned into droplets by using the QX200 Droplet Generator (Bio-Rad Laboratories, Hercules, CA, USA), transferred into a 96 well PCR plate and amplified according to manufacturer protocols. At the end of amplification reactions, fluorescent signals were measured with the QX200 Droplet Reader and analyzed using the QuantaSoft software version 1.7 (Bio-Rad Laboratories, Hercules, CA, USA).

Sensitivity and limit of detection (LOD) of ddPCR performed on DNA extracted from oral fluids has been determined by testing serial dilutions of mutant DNA and linear regression analysis, (**Supplementary Figure S1**, **S2**). Each dilution was run in duplicates and analyzed as a meta-well. The limit of blank (LOB) was calculated by determining the false-positive mean and the relative standard deviation of ddPCR assays in duplicates of genomic DNA (100ng) extracted from oral rinses from three healthy subject (**Supplementary Figure S4**). The thresholds for TERTp mut/TERTp wild-type positive events were set between 3500 and 2000 for each reaction. Then, the mutant alleles and wild-type allele numbers (copies/20μL) were used to calculate the mutant allele frequencies by using the formula MAF = Mut/(Mut + Wt). **Supplementary Table S1** provides the checklist for “Minimum Information for Publication of Quantitative Digital PCR Experiments for 2020 (dMIQE2020)” applied to ddPCR amplification of DNA extracted from oral fluids (23). Specificity, sensitivity and limit of detection (LOD) of ddPCR assays performed on DNA extracted from FFPE tissues has been previously established by testing mutant and wild-type templates by an orthogonal method (24).

Datasets are available at https://zenodo.org/records/15394798 (DOI 10.5281/zenodo.15394797).

### TERT gene expression by real-time PCR

For each cell line, 500 ng of total RNA were reverse transcribed in 20 μL volume containing 4 μL of iScript reaction mix, 1 μL of iScript reverse transcriptase (Bio-Rad Laboratories Inc., Hercules, California, U.S.A.) and incubated at 25°C for 5 min, at 42°C for 30 min and at 85°C for 5 min in a Mastercycler X50s (Eppendorf, Hamburg, Germany) thermal cycler. TERT expression levels were quantified by real-time PCR using forward (5′-CGGAAGAGTGTCTGGAGCAA-3′) and reverse (5′-GGATGAAGCGGAGTCTGGA-3′) primers designed to amplify regions within exons 3 and 4 of the TERT gene. This primer set spans intron 3, thus preventing amplification from potential genomic DNA contamination. Each PCR reaction contained 12.5 μL of 1× iQ™ SYBR^®^ Green Supermix (Bio-Rad Laboratories Inc.), 10 pmol of each primer, 1 μL of cDNA template, and nuclease-free water to a final volume of 25 μL. All reactions were performed in duplicate using the CFX96 Real-Time PCR Detection System (Bio-Rad Laboratories Inc.). Gene expression levels were quantified using the 2^−^ΔCt method, with GAPDH serving as the internal reference. For each target transcript, ΔCt values were obtained by subtracting the GAPDH Ct value from the corresponding gene Ct value (ΔCt = Ctx − CtGAPDH). To enable accurate comparison across different genes, Ct values were corrected based on primer pair efficiencies.

### Statistical analysis

Statistical analyses were performed using R packages and GraphPad Prism version 8.3.0. The prevalence of TERTp mutations was estimated as the proportion of oral rinses and FFPE tissue samples that tested positive for TERTp mutations C228T or C250T by ddPCR assays. Logistic regression models were performed to evaluate association between TERTp status and patient variables. Mantel Haenszel corrected χ^2^ test or fisher’s exact test, as appropriate, were used to define statistical significance of differences between categorical variables. Wilcoxon rank-sum test was used for comparisons between continuous variables. Differences were considered statistically significant when P values were less than 0.05. The accuracy, sensitivity and specificity of TERTp mutations testing in oral fluids and FFPE tumor tissues were calculated according to conventional formulas.

## Results

### Characteristics of HNSCC patients

The study included a cohort of 132 patients diagnosed with HNSCC and nine with non-HNSCC lesions. Demographic and clinic-pathologic data for all HNSCC patients participating in this study are summarized in **Table 1**. The mean age at diagnosis was 63.3 years (SD ± 11.7), with male patients significantly younger (mean age 63.1 years, SD ± 9.5) than female patients (mean age 67 years, SD ± 13.5), P = 0.035. Moreover, males accounted for the majority of HNSCC cases (73%) while females represented 27%, with the exception of the oral cancer subgroup, where a similar distribution of OSCC cases was observed between males (54%) and females (46%).

**Table 1:** Clinical characteristics of patients enrolled in the study.

Oral samples	Total (N = 132)
Median age at diagnosis, years (IQ range)	65 (56 - 72)
Sex (%)	
Female	36 (27%)
Male	96 (73%)
Site (%)	
Hypopharynx	10 (8%)
Larynx	45 (34%)
Oral cavity	50 (38%)
Oropharynx	25 (19%)
Unknown	2 (2%)
Tumor Clinical Stage (%)	
cT1	31 (23.5%)
cT2	29 (22.0%)
cT3	40 (30.3%)
cT4A	28 (21.2%)
cT4B	2 (1.5%)
Missing	2 (1.5%)
Smoke (%)	
Never	40 (30%)
Active, former	92 (70%)
Oral high-risk HPV status (%)*	
Negative	107 (81%)
Positive	25 (19%)
Oral TERTp mutation status (%)	
Mutant	33 (25%)
Wild type	99 (75%)

*Reported in Galati et al. (5).

Regarding tumor site, the oral cavity was the most frequently affected location (38%), followed by the larynx (34%), the oropharynx (19%), and the hypopharynx (8%). Clinical staging was assigned according to the 8th Edition of the TNM classification of malignant tumors (25). HNSCC patients were classified as clinical stage T1 (cT1 = 31, 22%), cT2 (n=29, 22%), cT3 (n=40, 30%), cT4A (n=28, 21%), and cT4B (n=2, 1.5%), (**Table 1**).

Among the 132 patients, 40 (30%) were non‐smokers, 50 (38%) were former smokers, who quit smoking at least 12 months before diagnosis, and 42 (32%) were active smokers. In the latter patient group, 2.4% (1 out of 42) smoked less than 10 pack/year, 24% (10 out of 42) smoked between 10 and 20 pack/year, and 43 (91%) were heavy smokers with a smoking history above 20 pack/year. In the group of the former smokers, 1 (2%) smoked less than 10 pack/year, 12 (23%) between 10 and 20 pack/year and 39 (75%) more than 20 pack/year.

Total DNA extracted from oral rinses (n=132) and FFPE tumor tissues (n=63) was previously analyzed for the presence of high-risk HPV (5). The results showed that 21% of oral rinses and 26% of tumor tissues tested positive for viral infection, with HPV16 as the most prevalent genotype, particularly in OPSCC cases in which HPV16 DNA was detected in 71% of tumor tissues.

### TERTp mutations in oral rinses

**Figure 1:**
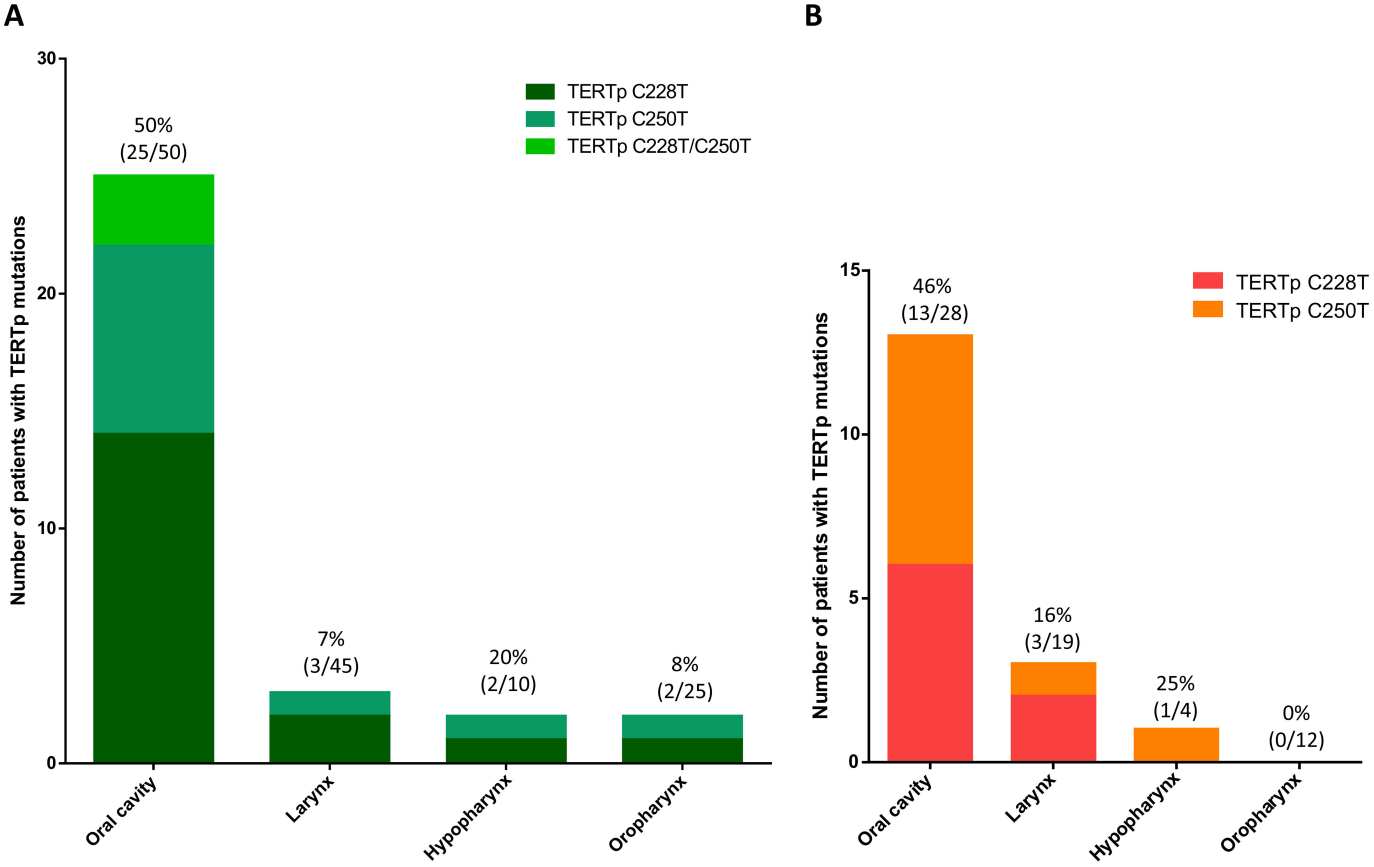
**(A)** Distribution of TERTp C228T and C250T as well as co-occurrence of C228T/C250T mutations in DNA extracted from oral rinses. TERTp mutations are significantly more frequent in oral cavity SCC than in larynx, hypopharynx and oropharynx SCC (P < 0.001). **(B)** Distribution of TERTp C228T and C250T mutations in DNA extracted from FFPE HNSCC tissues. TERTp C228T and C250T were similarly represented in OSCC tissues. No co-occurrence of TERTp C228T/C250T mutations was observed in HNSCC tissues.

A total of 132 oral fluid samples from HNSCC patients and nine oral samples from non-HNSCC patients have been analyzed for TERTp mutations C228T and C250T by ddPCR. TERTp C228T or C250T were identified in 33 out of 132 (25%) oral rinses from HNSCC patients and in one out of nine (10%) non-HNSCC patients (**Table 2**). Mutant TERTp C228T was the most prevalent being identified in 19 out of 33 (57.6%), while TERTp C250T was detect in 11 out of 33 (33.3%) and co-mutations C228T/C250T detected in three out of 33 (9.1%) TERTp mutants (**Figure 1**).

**Table 2:** Characteristics of HNSCC patients according to TERTp mutations in oral gargles.

Oral samples	TERTp mutant (N = 33)	TERTp wild type (N = 99)	p-value
Median age at diagnosis, years (IQR)	64 (56 - 73)	65 (56 - 71)	0.590
Sex (%)			
Female	12 (36%)	24 (24%)	0.270
Male	21 (64%)	75 (76%)	
Site (%)			
Hypopharynx	2 (6%)	8 (8%)	**<0.001**
Larynx	3 (9%)	42 (42%)	
Oral cavity	25 (76%)	25 (25%)	
Oropharynx	2 (6%)	23 (23%)	
Unknown	1 (3%)	1 (1%)	
Tumor clinical stage (%)			
cT1	0 (0%)	31 (31%)	**0.003**
cT2	11 (33%)	18 (18%)	
cT3	12 (36%)	28 (28%)	
cT4A	8 (24%)	20 (20%)	
cT4B	1 (3%)	1 (1%)	
Missing	1 (3%)	1 (1%)	
Smoke (%)			
Former/Active	16 (48%)	24 (24%)	**0.012**
Never	17 (52%)	75 (76%)	
Oral high-risk HPV status (%)			
Negative	28 (85%)	79 (80%)	0.620
Positive	5 (15%)	20 (20%)	

Significant p-values are indicated in bold.

Notably, TERTp mutations were significantly more frequent (25 out of 50, 50%, P = 0.001) in oral fluids from OSCC patients, mainly in those presenting with a stage cT2 (32%), cT3 (30%) and cT4 (28%), P = 0.003. The remaining TERTp mutations (n=8) were detected in LSCC (n=2 stage cT4 and n=1 cT1), OPSCC (n=1 cT1 and n=1 cT2), HPSCC (n=2 stage cT4) and in one unknown site SCC. Among the nine non-HNSCC oral rinse samples, only one obtained from a patient diagnosed with oral dysplasia, tested positive for the TERTp C228T mutation.

**Figure 2:**
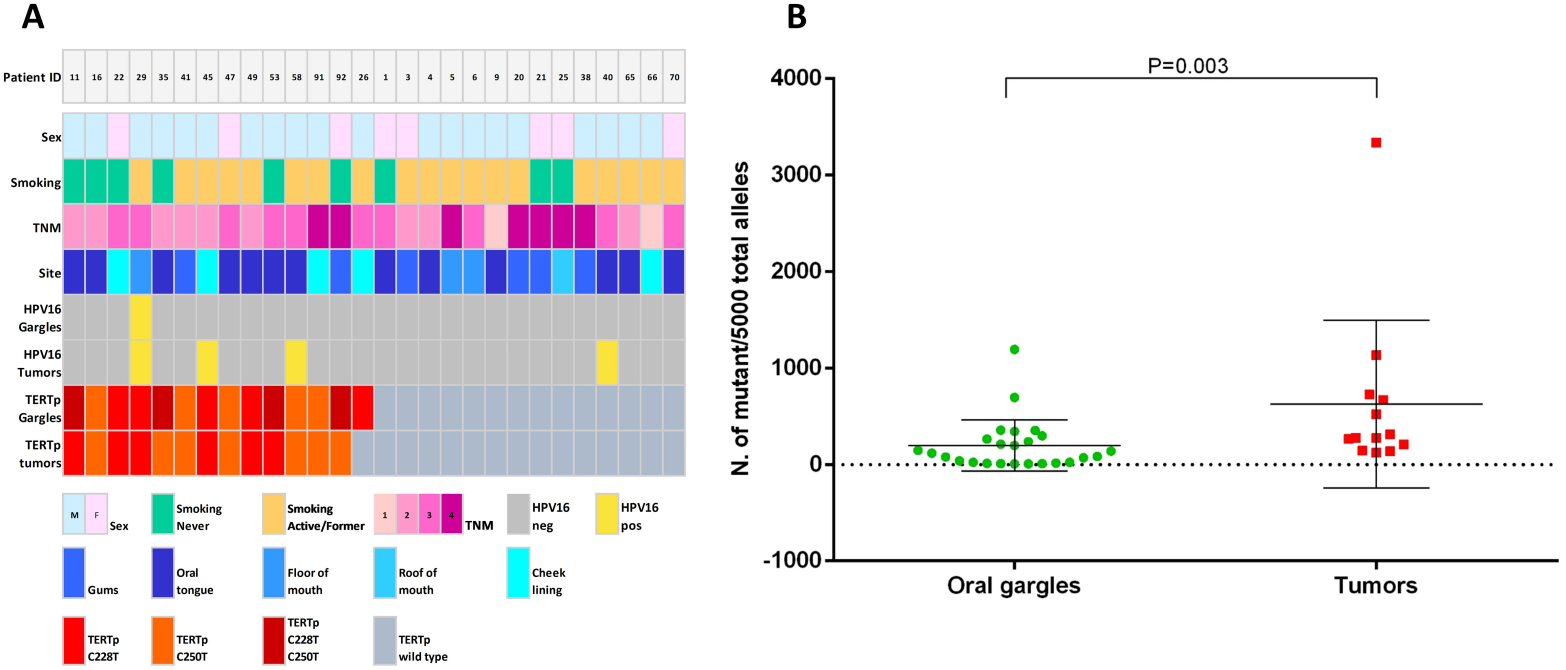
**(A)** Characteristics of 28 OSCC patients and TERTp mutation profile in their oral rinses and corresponding tumor tissues. **(B)** Quantification of TERTp mutant alleles in oral rinse samples (green dots) and FFPE OSCC tissues (red dots).

In oral rinses from OSCC patients the mutant allele frequencies (MAFs) of TERTp C228T or C250T ranged from 0.14% to 23.9%, with most values being lower than 10%, likely due to the high proportion of non-tumor cells exfoliated from the mucosa lining the head and neck region (**Figure 2**). In oral samples from non-OSCC patients the TERTp C228T and C250T MAFs were lower, ranging from 0.1% to 3.9%, suggesting that the site of head and neck cancers influence the release of tumor cells in the oral cavity and the possibility to collect them through gargling.

### TERTp mutations in HNSCC tissues

A total of 63 FFPE HNSCC tissues have been retrieved and analyzed for TERTp C228T and C250T nucleotide changes. TERTp mutations were identified in 25.4% (16 out of 63) DNA samples extracted from FFPE tumor tissues. Among them, the prevalence of TERTp mutations was 46% (13 out of 28) in OSCC, 16% (3 out of 19) in LSCC, 25% (1 out of 4) in HPSCC and no mutations in OPSCC (**Figure 1**). Among OSCC sub-sites, the mutation frequency of TERTp was remarkably higher (7 out of 13 cases, 54%) in oral tongue SCC compared to cancers arising in other oral sites (6 out of 15, 40%).

In OSCC tissues, the MAFs of TERTp mutations C228T and C250T ranged from 2.41% to 22.63%, with a single sample exhibiting a MAF of 66.75%, consistent with TERT gene amplification (**Figure 2**). On the other hand, the MAF of TERTp mutations in non-OSCC tumors ranged from 0.3% to 17.9%, with most cases exhibiting frequencies below 10%.

### Correlation of TERTp mutations between oral fluids and tumor tissues

To assess the accuracy of TERTp mutations in oral rinses for non-invasive cancer detection in HNSCC patients, we evaluated whether mutations identified in oral rinse-derived DNA were also present in matched tumor tissues. The limit of detection of TERTp mutations by ddPCR in our study was ≥0.1% and the overall concordance of TERTp mutations between oral fluids and HNSCC across all tumor sites was 89% (95%CI 78.4–95.4), (**Supplementary Table S1**). Given the significantly higher rate of TERTp mutations in both oral samples and tumor tissues from OSCC patients, we restricted our analysis to the oral cancer subgroup (**Table 3**). The intra-patient concordance of TERTp mutations between oral rinse specimens and OSCC tissues was 93% (95%CI 76.5 – 99.1), with a sensitivity of 86.7% (95%CI 59.54 - 98.3%) and specificity of 100% (95%CI 75.3 – 100). On the other hand, concordance between oral samples and tumor tissues in non-OSCC cases was 85.7% (95%CI 69.7 – 95.2) with a sensitivity of 40% (95%CI 5.3 - 85.3) and specificity of 93% (95%CI 77.9 - 99.2). TERTp mutations were specific to neoplastic cells since oral rinses DNA from non-HNSCC patients were found not mutated, except for one patient diagnosed with oral dysplasia.

**Table 3:** Patient characteristics according to tumor site (OSCC versus non-OSCC).

Oral samples	Non-OSCC (N = 82)	OSCC (N = 50)	p-value
Median age at diagnosis, years (IQR)	65 (58 - 69)	65 (53 - 75)	0.45
Sex (%)			
Female	13 (16%)	23 (46%)	**<0.001**
Male	69 (84%)	27 (54%)	
Tumor clinical stage (%)			
cT1	26 (32%)	5 (10%)	**0.009**
cT2	13 (16%)	16 (32%)	
cT3	25 (30%)	15 (30%)	
cT4A	14 (17%)	14 (28%)	
cT4B	2 (2%)	0 (0%)	
Missing	2 (2%)		
Smoke (%)			
Former/Active	19 (24%)	21 (42%)	**0.031**
Never	63 (76%)	29 (58%)	
Oral high-risk HPV status (%)			
Negative	62 (76%)	45 (90%)	0.062
Positive	20 (24%)	5 (10%)	
Oral TERTp mutation status (%)			
Mutant	8 (10%)	25 (50%)	**<0.001**
Wild-type	74 (90%)	25 (50%)	

Significant p-values are indicated in bold.

Taken together, our data support the feasibility of reliable identification of TERTp mutations in oral rinse samples to diagnose OSCC, even at early stages (cT2) of the disease (**Supplementary Table S2**).

### TERT promoter mutations and TERT gene expression levels in oral cancer cell lines

**Figure 3:**
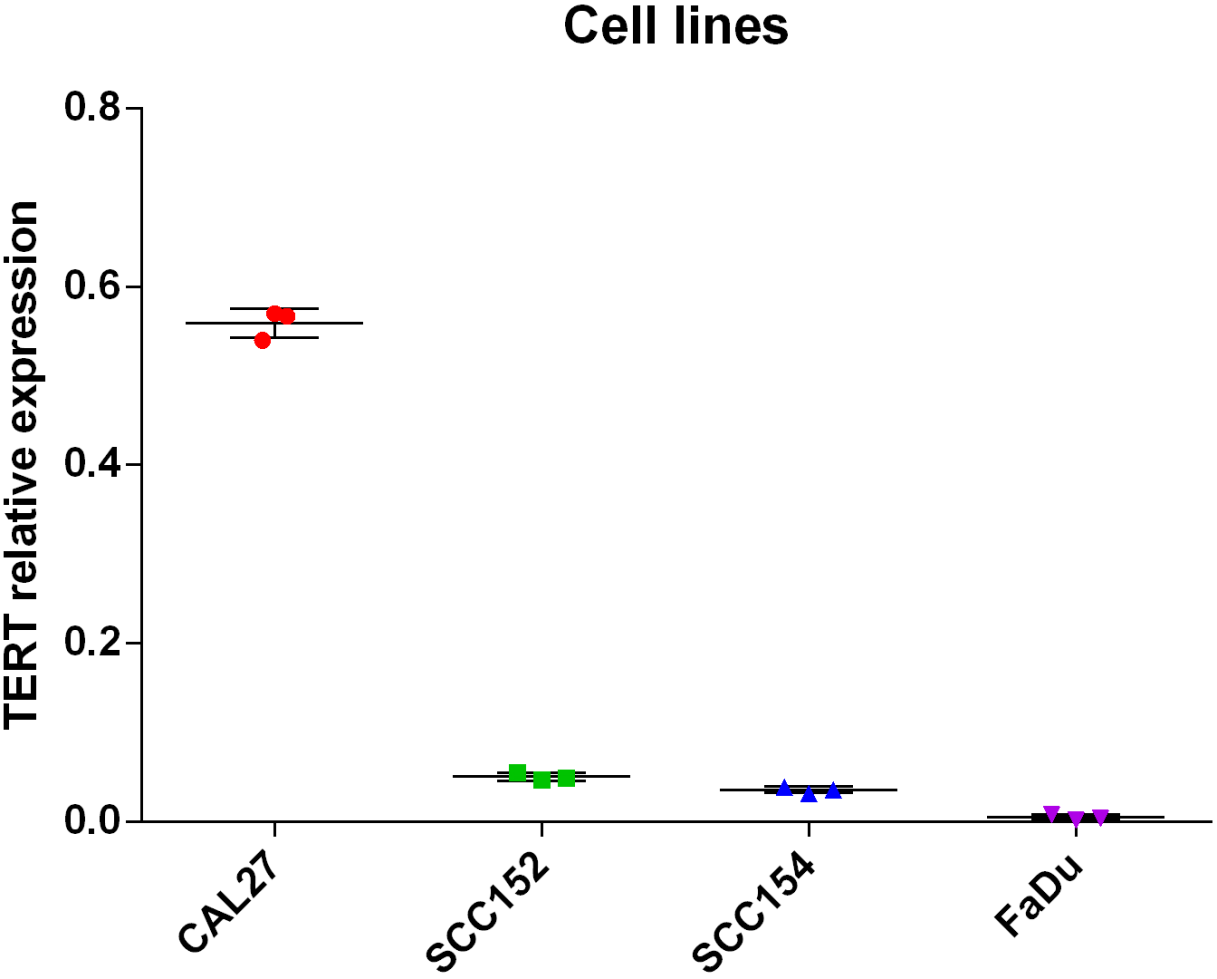
The relative expression values, normalized to GAPDH mRNA, of TERT mRNAs in the relative expression values, normalized to GAPDH mRNA, of TERT mRNAs in HPV-negative/TERTp C250T (CAL27), HPV-positive TERTp wild-type (SCC152 and SCC154) and HPV-negative TERTp wild-type (FaDu) squamous cell carcinoma cell lines.

The analysis of TERTp mutations in DNA extracted from three oral squamous cell carcinoma derived cell lines, including HPV-negative (CAL27) and HPV16-positive (SCC152 and SCC154) as well as the HPV-16 hypopharyngeal squamous cell carcinoma derived cell line FaDu, revealed a C228T transition in the CAL27 cells (**Supplementary Table S3**, **Supplementary Figure S5**). Quantitative RT-PCR analysis of TERT mRNAs showed that CAL27 cells harboring this mutation exhibited 11-fold and 15-fold increase compared to HPV16 positive TERTp wild-type SCC152 and SCC154 cells, respectively, and above 100-fold increase versus HPV negative TERTp wild-type FaDu cells (**Figure 3**). These results demonstrate that the TERTp C228T mutation exerts a much stronger effect than the HPV16 E6 protein on telomerase activation.

## Discussion

Somatic mutations in the TERTp region have been reported at varying frequencies across different tumor types (16, 26). Within the heterogeneous group of head and neck cancers, the prevalence and clinical implications of TERTp mutations differ by anatomical site, being common in OSCC but infrequent in HNSCC originating from sites other than oral cavity, such as such as the oropharynx, larynx, and hypopharynx (19).

In our study we investigated the distribution of TERTp mutations in FFPE tumor tissues and matched oral rinses from patients diagnosed with HNSCC at different sites. The results confirmed previous data on the high prevalence of such mutations in OSCC (46%) and lower frequency in LSCC (16%) and HPSCC (25%). Unlikely other tumor types, such as hepatocellular carcinoma in which TERTp C228T represents 95% of all TERTp mutations (27), both hot spot TERTp C228T and C250T, were similarly distributed in OSCC, with C228T being only slightly more frequent than C250T. Such results suggest that detection of TERTp C228T and C250T mutations in oral fluids could serve as potential biomarker for the diagnosis of a significant number of OSCC.

The prevalence of TERTp mutations in OSCC exhibits significant geographic variation. A recent systematic review including 816 OSCC cases across 13 studies reported an average frequency of TERTp mutations of 46% (28). However, the analysis revealed considerable heterogeneity in the distribution of mutations among different populations, with the highest prevalence observed in Asian OSCC patients (70%) (29). In Italy, two independent studies reported comparable frequencies of TERTp mutations in OSCC, with prevalence of 33% in the Campania region and 31% in the Veneto region (18, 30). These rates are lower than the 46% mutation frequency observed in the present study of OSCC patients from the Lombardia region (this study). However, this difference may be attributable to the higher sensitivity of the mutation detection methods employed, with ddPCR having greater sensitivity compared to Sanger sequencing technique (24, 31).

The distribution of TERTp mutations has been shown to differ by sex across different cancer types. El Aziz et al. (2024) reported that the frequency of TERTp mutations in HNSCC was significantly higher in white females (50%) compared to white males (37%) patients (29). In our study, although females (46%) and males (54%) were quite similarly represented in the OSCC cohort, TERTp mutations were more frequently observed in males (57%) than in females (39%). This discrepancy may be partially explained by the higher prevalence of smoking habit among males (79%) compared to females (37%), suggesting a major role of tobacco exposure in the occurrence of TERTp mutations. This hypothesis is further supported by the significant difference in smoking habits between non-OSCC (24% former/active smokers) and the OSCC (42% former/active smokers) group. Accordingly, higher frequency of TERTp mutation C228T OSCC has shown to be correlated with betel nut chewing (32). Conversely, Arantes et al. (2020) reported that 94.4% of the patients harboring TERTp mutation C250T were alcohol consumers while 66.7% of the patients harboring C228T were non-alcohol consumers, in a cohort with 74.4% of alcohol consumers patients (33).

Cell lines originating from tumor types with a high prevalence of TERTp mutations often carry these alterations (34). Whole-genome and RNA sequencing of a broad panel of cancer cell lines revealed 60 lineages (18.23%) harboring TERT promoter mutations, all of which exhibited monoallelic TERT mRNA expression driven by the mutated TERTp (34). We have previously shown that TERT mRNA expression was markedly higher in cervical squamous cell carcinoma derived SiHa cells carrying TERTp C250T compared to TERTp wild type cervical cell lines further indicating that such mutations play a major role in TERT activation (18). In this study, we examined squamous carcinoma-derived cell lines, including the HPV-negative CAL27, as well as the HPV-positive SCC152, SCC154, and FaDu. The TERTp hotspot mutation C228T was identified exclusively in CAL27 cells, which are derived from a tongue squamous cell carcinoma (35). Notably, TERT mRNA levels were markedly higher in CAL27 cells compared to the other cell lines, regardless of HPV status, indicating that this promoter mutation is a key driver of TERT activation.

In recent years, viral circulating tumor DNA in virus-driven malignancies, such as HPV in oropharyngeal and cervical cancers, has been extensively investigated as a biomarker to monitor disease progression and recurrence. On the other hand, in HPV-negative HNSCC, tumor-informed circulating DNA assays based on detection of recurrent somatic alterations, such as TERTp mutations, offer a promising complementary strategy. Several studies have shown that TERTp mutations are associated with an increased risk of loco-regional failure in OSCC and remain an independent predictor in multivariate analyses (36). These findings suggest that TERTp mutations may define a high-risk molecular subset of OSCC characterized by distinct clinical behavior (37). While the development of targeted therapies against telomerase and TERTp mutations remains in its infancy, incorporating TERTp mutation testing into clinical practice could pave the way for precision medicine approaches, offering tailored treatment options and improving patient outcomes. In addition, detection of TERTp mutations in tumor DNA shed in the oral rinse could be emerging biomarkers for early detection of OSCC, risk-adapted surveillance for identification of minimal residual disease. Further research, including large-scale validation studies, will be essential to confirm the utility of TERTp assays in routine clinical practice, but their integration holds significant promise for advancing personalized oncology in head and neck cancer.

In our study there are several limitations. First, the number of cases included in the molecular analyses was limited. Second, the TERTp mutant detection with high sensitivity by ddPCR has been performed only for two hotspot mutations, therefore the total number of mutations in this genetic locus may be underestimated. Third limitation is related to the retrospective nature of the study that did not allow to evaluate the correlation between TERTp mutations and the clinical outcome in terms of progression free survival or overall survival. However, this is the first study to demonstrate that tumor DNA shed into saliva reflects the TERTp signature of the tumor, enabling non-invasive monitoring of tumor development and treatment response.

## Conclusions

Our results suggest that TERT promoter mutations are highly prevalent in OSCC, particularly among current and former smokers. These mutations can be reliably detected in tumor DNA released in the oral rinse samples with high sensitivity and specificity, highlighting their potential as non-invasive biomarkers for early OSCC detection. Further large-scale, prospective studies are needed to validate the identification of TERTp mutations in oral rinses as diagnostic biomarkers and to explore their potential as early detection of recurrence in oral cancer.

## Data Availability

The datasets presented in this study can be found in online repositories. The names of the repository/repositories and accession number(s) can be found below: https://zenodo.org/records/15394798.
