# Supplementary material for: Quantifying TERT promoter mutations in tumor-derived DNA shed into the oral cavity as a potential biomarker for oral squamous cell carcinoma
Source: Front Oncol. 2025 Dec 19;15:1720783. doi: 10.3389/fonc.2025.1720783 (PMC12757288; doi:10.3389/fonc.2025.1720783)
Supplement: Supplementary file 1 [file DataSheet1.pdf]

Quantification of TERT promoter mutations in tumor DNA shed into the oral cavity as a potential biomarker for oral squamous cell carcinoma by *Starita et al.*

Table 1S. dMIQE2020 checklist for TERTp C228T/C250T ddPCR assays.

ITEM TO CHECK	**PROVIDED**	**COMMENT**
	**Y/N**	
1. SPECIMEN		
Detailed description of specimen type and numbers	Y	Gargle specimens from patients diagnosed with HNSCC (N=132)
Sampling procedure (including time to storage)	Y	Patients provided oral samples by gargling 15 mL of 0.9% NaCl sterile solution in the oral cavity for 15 seconds.
Sample aliquotation, storage conditions and duration	Y	Oral fluid samples stored at -20°C until analysis
2. NUCLEIC ACID EXTRACTION		
Description of extraction method including amount of sample processed	Y	Genomic DNA was extracted from oral fluids using the BioRobot EZ1 (Qiagen, Hilden, Germany) Briefly, samples were centrifuged at 6,000 rpm for 10 minutes, cell pellets were resuspended in the buffer G2 containing proteinase K and incubated at 56°C for 3 hours. Purified DNA was eluted into 50µL of elution buffer.
Volume of solvent used to elute/resuspend extract	Y	50µL elution buffer
Number of extraction replicates	Y	1-2
Extraction blanks included?	Y	Extraction of blanks are included every 5 samples
3. NUCLEIC ACID ASSESSMENT AND STORAGE		
Method to evaluate quality of nucleic acids	Y	See below
Method to evaluate quantity of nucleic acids (including molecular weight and calculations when using mass)	Y	Quantity of DNA samples are determined by Nanodrop 2000c spectrophotometer (Thermo Fisher Scientific, Waltham, Massachusetts). Quality is assessed by the ratio of absorbance at 260 nm and 280 nm required to be above 1.8
Storage conditions: temperature, concentration, duration, buffer, aliquots	Y	3-5 aliquots of each DNA sample (50ng/µL TE) are stored at -80°C for two years
Clear description of dilution steps used to prepare working DNA solution	Y	Working DNA solution (50ng/µL) is obtained by calculating the dilution factor ([DNA]ng/50ng) and by adding the stock DNA to the required TE volume.
4. NUCLEIC ACID MODIFICATION		
Template modification (digestion, sonication, pre-amplification, bisulphite etc.)	N	
Details of repurification following modification if performed	N	
5. REVERSE TRANSCRIPTION		
cDNA priming method and concentration		
One or two step protocol (include reaction details for two step)	N	
Amount of RNA added per reaction	N	
Detailed reaction components and conditions	N	
*Estimated copies measured with and without addition of RT**	N	
Manufacturer of reagents used with catalogue and lot numbers	N	

Storage of cDNA: temperature, concentration, duration, buffer and aliquots	N	
6. ddPCR PRIMERS DESIGN AND TARGET INFORMATION		
Sequence accession number or official gene symbol	Y	dHsaEXD72405942 (TERT C228T_113); dHsaEXD46675715 (TERT C250T_113)
Method (software) used for design and in silico verification	N	
Location of amplicon	N	
Amplicon length	Y	113 bp
*Primer and probe sequences (or amplicon context sequence)***	N	
Location and identity of any modifications	N	
Manufacturer of oligonucleotides	Y	Bio-Rad Laboratories, USA
7. ddPCR PROTOCOL		
Manufacturer of ddPCR instrument and instrument model	Y	QX200 Digital droplet PCR System, Bio-Rad Laboratories
Buffer/kit manufacturer with catalogue and lot number	Y	ddPCR Supermix for Probes (No dUTP) #1863024
Primer and probe concentration	N	
Pre-reaction volume and composition (incl. amount of template and if restriction enzyme added)	Y	20µl final volume comprising:<br>10µl (2x) ddPCR Super Mix;<br>1µl (20x) TERT C228T_113 assay or TERT C250T_113 assay;<br>2µl of 5M Betaine (Sigma Aldrich);<br>0.25µl of 80mM EDTA;<br>100ng DNA;<br>deionized distilled water to 20µl.<br>No addition of restriction endonuclease.
Template treatment (initial heating or chemical denaturation)	N	
*Polymerase identity and concentration, Mg++ and dNTP concentrations****	N	
Complete thermocycling parameters	Y	1. Enzyme activation: 10 min 95˚C;<br>2. 50 cycles of 1 min annealing/ extension at 62˚C and 30 sec denaturation at 96˚C;<br>3. Enzyme deactivation: 10 min at 98˚C<br>4. Rate of temperature rise 2.5˚C/sec.<br>5. Holding at 4 ˚C.
8. ASSAY VALIDATION		
Details of optimisation performed	N	
Analytical specificity (vs. related sequences) and limit of blank (LOB)	Y	Figure 4S
Analytical sensitivity/LOD and how this was evaluated	Y	Figure 1S, Figure 2S, Figure 3S
Testing for inhibitors (from biological matrix/extraction)	N	
9. DATA ANALYSIS		
Description of ddPCR experimental design	Y	See material and Methods
Comprehensive details negative and positive of controls (whether applied for QC or for estimation of error)	Y	Figure 1S, Figure 2S, Figure 3S and Figure 4S

Quantification of TERT promoter mutations in tumor DNA shed into the oral cavity as a potential biomarker for oral squamous cell carcinoma by *Starita et al.*

Partition classification method (thresholding)	N	Manual
Examples of positive and negative experimental results (including fluorescence plots in supplemental material)	Y	Figure 1S, Figure 3S
Description of technical replication	Y	Figure 1S, Figure 2S, Figure 3S, Figure 4S
Repeatability (intra-experiment variation)	Y	Figure 1S, Figure 2S, Figure 3S, Figure 4S
Reproducibility (inter-experiment/user/lab etc. variation)	Y	Figure 1S, Figure 2S, Figure 3S, Figure 4S
Number of partitions measured (average and standard deviation)	Y	18,869 (±2078)
Partition volume	N	
Copies per partition (λ or equivalent) (average and standard deviation)	N	
ddPCR analysis program (source, version)	Y	See Material and Methods
Description of normalisation method	N	
Statistical methods used for analysis	Y	See Material and Methods
Data transparency	raw data available on request	m.tornesello@istitutotumori.na.it

* Assessing the absence of DNA using a no RT assay (or where RT has been inactivated) is essential when first extracting RNA. Once the sample has been validated as DNA-free, inclusion of a no-RT control is desirable, but no longer essential.

** Disclosure of the primer and probe sequence is highly desirable and strongly encouraged. However, since not all commercial pre-designed assay vendors provide this information when it is not available assay context sequences must be submitted (Bustin et al. Primer sequence disclosure: A clarification of the MIQe guidelines. Clin Chem 2011;57:919-21.)

*** Details of reaction components is highly desirable, however not always possible for commercial disclosure reasons. Inclusion of catalogue number is essential where component reagent details are not available.

Quantification of TERT promoter mutations in tumor DNA shed into the oral cavity as a potential biomarker for oral squamous cell carcinoma by *Starita et al.*

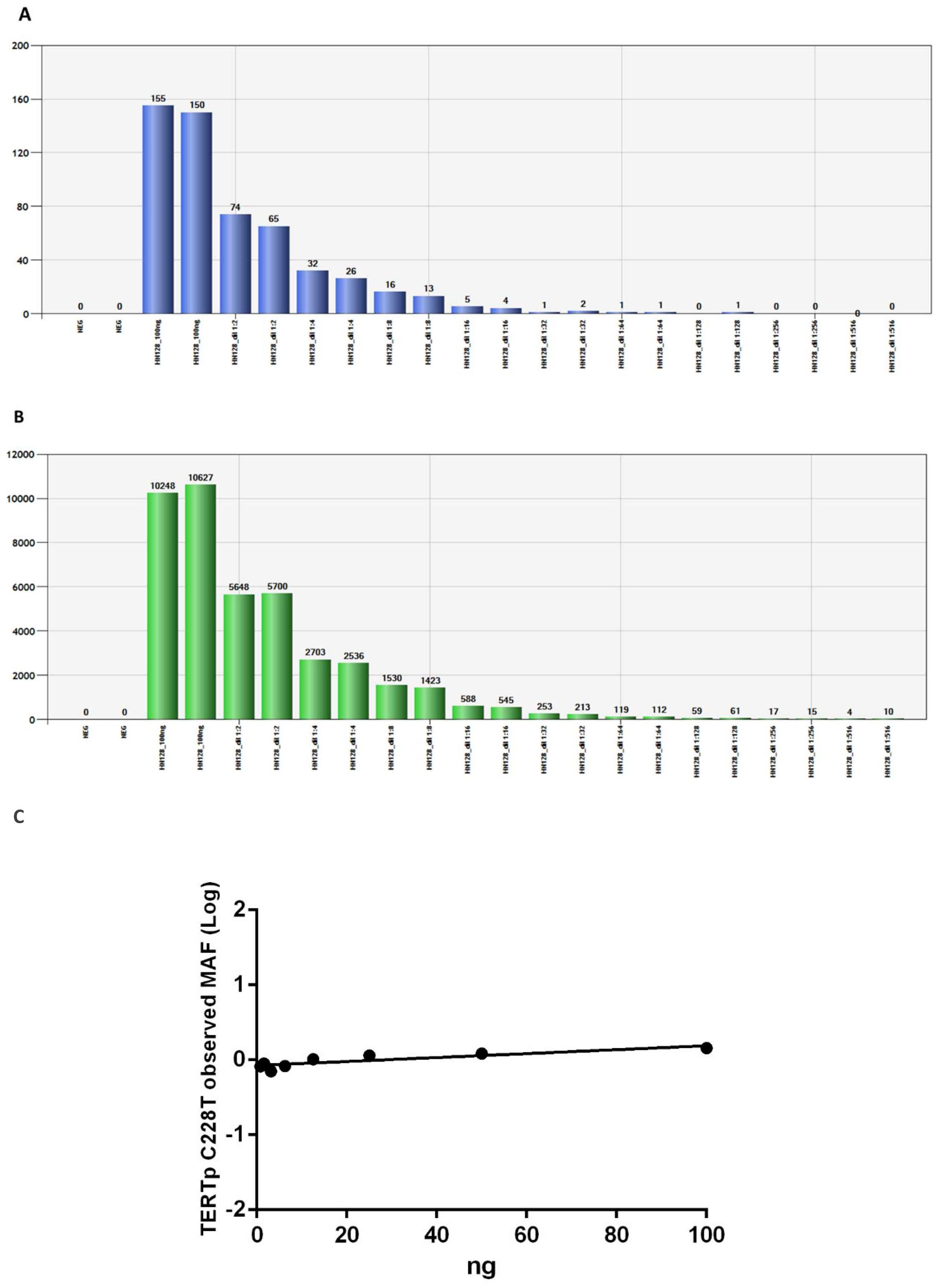

Figure 1S. Assessment of linear range and precision of ddPCR for low DNA target extracted from oral gargles. Observed number of mutant and wild type allele in serial dilutions (1:2) from 100 ng down to 0.2 ng of sample HN128. Number of TERTp C228T (A) and TERTp WT (B) in each reaction. (C) Observed MAFs in serial dilutions of C228T mutant sample.

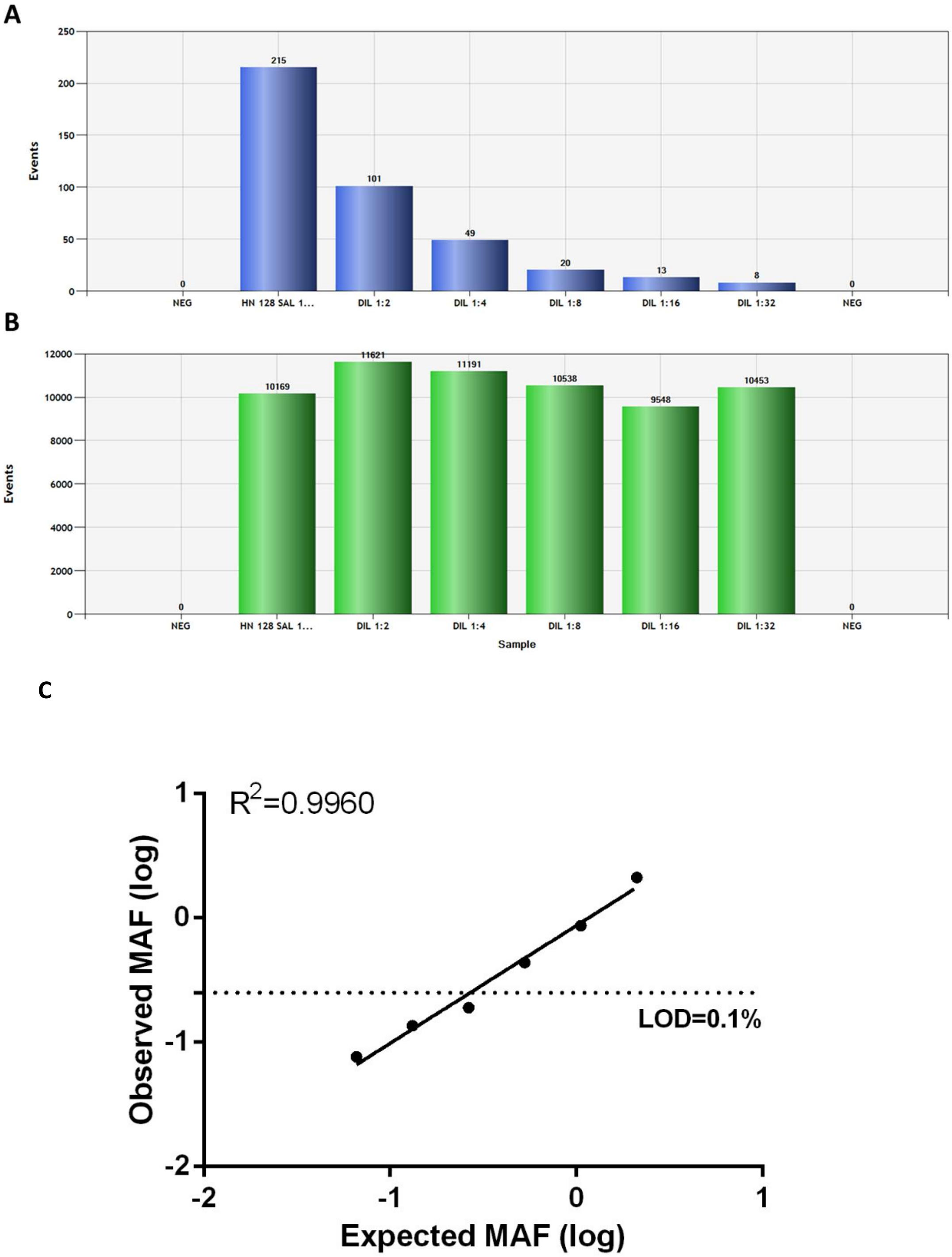

Figure 2S. Limit of detection (LOD) for TERTp C228T in DNA extracted from oral fluid sample and diluted in 100ng TERTp wild type DNA extracted from HeLa cells. Observed versus expected TERTp mutant events in serial dilutions (1:2) of HN128. Limit of detection (LOD) = 0.1%. Number of TERTp C228T (A) and TERTp WT (B) in each reaction. (C) Observed MAFs in serial dilutions of C228T mutant sample.

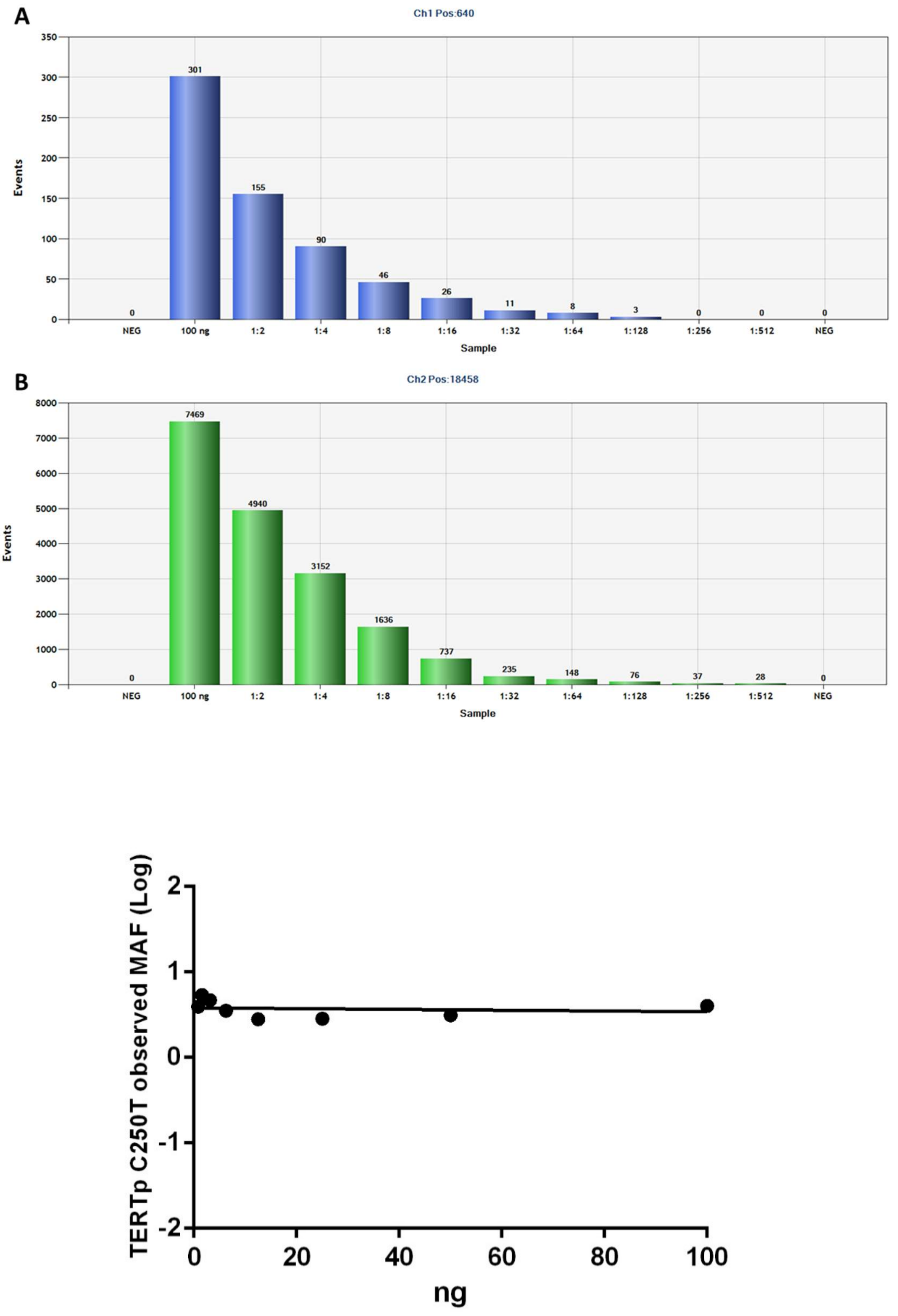

Figure 3S. Assessment of linear range and precision of ddPCR for low DNA target extracted from oral gargles. Observed number of mutant and wild type allele in serial dilutions (1:2) from 100 ng down to 0.2 ng of sample HN137. Number of TERTp C250T (A) and TERTp WT (B) in each reaction. (C) Observed MAFs in mutant serial dilutions.

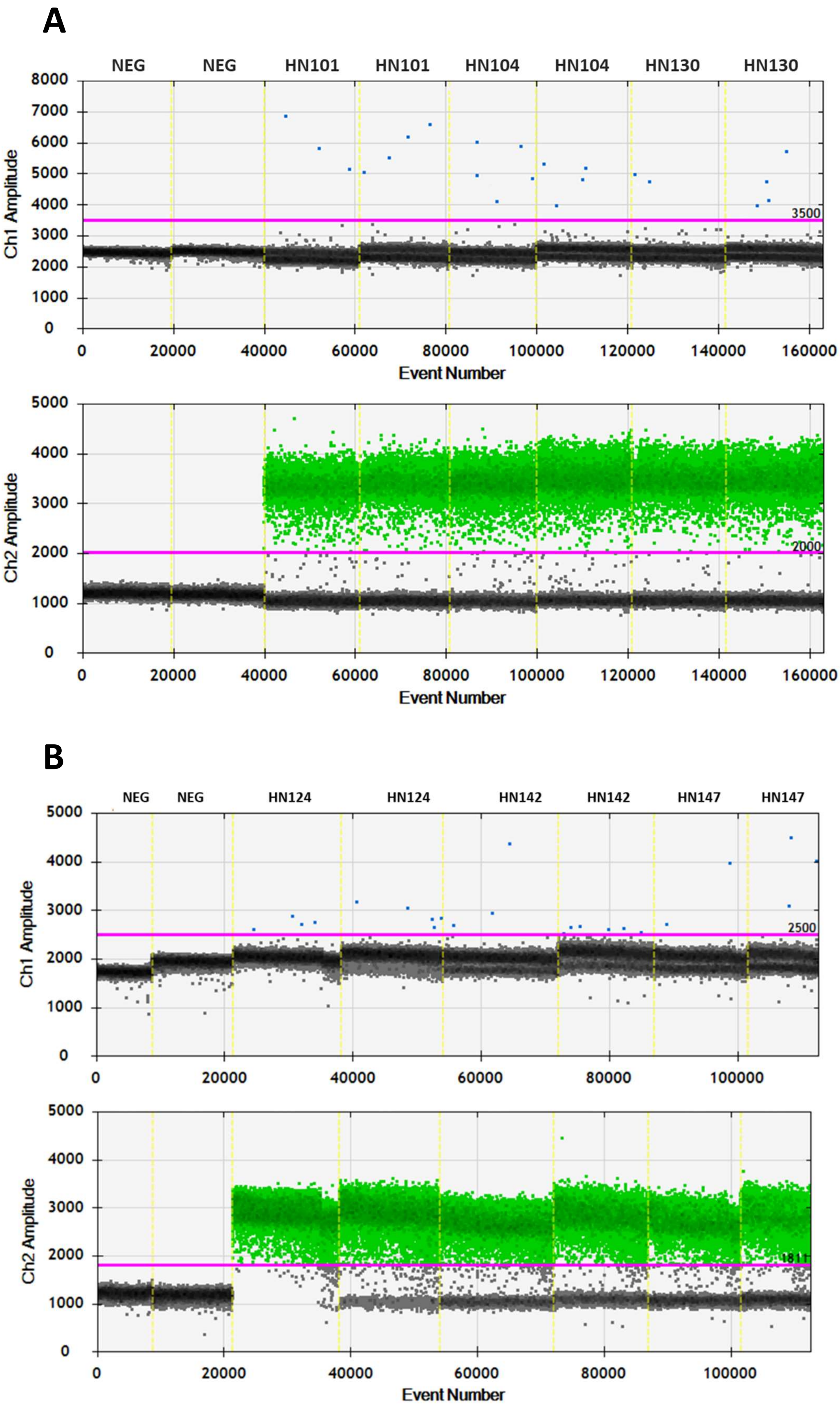

Figure 4S. False-positive evaluation and limit of blank (LOB) for C228T TERTp (A) and C250T TERTp (B) assays in genomic DNA extracted from three healthy subject (negative controls). The DNA target was 100ng for each sample. The mean number of false positive events was 3.7 (±1.0).

Table 2S. Clinical characteristics, smoking history, HPV status and TERTp mutations, detected by ddPCR, in DNA samples extracted from oral gargles and tumour tissues of HNSCC patients

ID	Sex	Age	Smoking* (p/y)	Site	Stage TNM	HPV16** Gargle	HPV16** Tumor	TERTp Gargle (MAF)	TERTp Tumor (MAF)
1	F	42	Never	Oral cavity	3	0	0	wt	wt
3	F	75	Former (46 p/y)	Oral cavity	2	neg	neg	wt	wt
4	M	80	Active (20 p/y)	Oral cavity	2	neg	neg	wt	wt
5	M	39	Active (22 p/y)	Oral cavity	4a	neg	neg	wt	wt
6	M	68	Former (40 p/y)	Oral cavity	3	neg	neg	wt	wt
9	M	72	Former (25 p/y)	Oral cavity	1	neg	neg	wt	wt
20	M	63	Active (35 p/y)	Oral cavity	4a	neg	neg	wt	wt
21	F	82	Never	Oral cavity	4a	neg	neg	wt	wt
25	F	77	Never	Oral cavity	4a	neg	neg	wt	wt
26	M	68	Active (45 p/y)	Oral cavity	3	neg	neg	C228T (2.95%)	wt
11	M	50	Never	Oral cavity	2	neg	neg	C228T/C250T (0.14%, 0.2%)	C228T (2.82%)
16	M	57	Never	Oral cavity	2	neg	neg	C250T (0.15%)	C250T (5.29%)
38	M	62	Active (45 p/y)	Oral cavity	4a	neg	neg	Wt	wt
40	M	42	Former (12 p/y)	Oral cavity	3	0	HPV16 (683)	Wt	wt
22	F	68	Never	Oral cavity	3	0	0	C228T (4.22%)	C228T (22.63%)
29	M	75	Former (15 p/y)	Oral cavity	3	HPV16	HPV16	C228T (0.33%)	C228T (6.21%)
35	M	72	Never	Oral cavity	2	0	0	C228T/C250T (0.41%, 2.35%)	C250T (4.14%)
41	M	61	Former (20 p/y)	Oral cavity	2	0	0	C250T (0.14%)	C250T (13.40%)
45	M	63	Former (20 p/y)	Oral cavity	2	0	HPV16	C228T (5.29%)	C228T (2.41%)
47	F	49	Former (20 p/y)	Oral cavity	3	0	0	C250T (0.18%)	C250T (5.48%)
49	M	52	Former (30 p/y)	Oral cavity	2	0	0	C228T (0.28%)	C228T (5.55%)
53	M	46	Never	Oral cavity	3	0	0	C228T/C250T (0.80%, 0.15%)	C228T (2.76%)
58	M	36	Former (30 p/y)	Oral cavity	3	0	HPV16	C250T (1.59%)	C250T (10.44%)
65	M	35	Former (15 p/y)	Oral cavity	2	0	0	wt	wt
66	M	53	Active (35 p/y)	Oral cavity	1	0	0	wt	wt
70	F	42	Active (20 p/y)	Oral cavity	3	0	0	wt	wt
91	M	53	Active (30 p/y)	Oral cavity	4a	0	0	C250T (1.44%)	C250T (66.75%)
92	F	84	Never	Oral cavity	4a	0	0	C228T/C250T (0.14%, 5.97%)	C250T (14.51%)
18	F	56	Active (35 p/y)	Hypopharynx	4b	0	0	C250T (0.14%)	C250T (8.92%)

13	M	55	Active (40 p/y)	Hypopharynx	4a	0	0	wt	wt
14	M	59	Active (20 p/y)	Hypopharynx	1	0	0	wt	wt
69	M	50	Active (30 p/y)	Hypopharynx	4a	0	0	C228T (0.87%)	wt
12	M	65	Former (40 p/y)	Larynx	3	0	0	wt	wt
44	M	69	Former (30 p/y	Larynx	1	0	HPV16	wt	C250T (17.94%)
17	M	51	Active (45 p/y)	Larynx	4a	0	0	wt	wt
51	F	68	Active (45 p/y	Larynx	3	0	0	wt	C250T (1.82%)
19	M	72	Never	Larynx	4a	0	0	wt	wt
63	M	68	Active (45 p/y)	Larynx	3	0	0	C228T (0.14%)	C228T (0.33%)
23	M	81	Never	Larynx	4a	HPV16	HPV16	C250T (0.24%)	wt
30	M	51	Former (20 p/y)	Larynx	3	0	HPV16	wt	wt
34	M	58	Former (42 p/y)	Larynx	1	0	0	wt	wt
37	M	26	Former (6 p/y)	Larynx	3	0	0	wt	wt
39	M	71	Former (15 p/y)	Larynx	2	0	HPV16	wt	wt
43	F	59	Never	Larynx	4a	0	0	wt	wt
48	M	65	Never	Larynx	3	0	0	wt	wt
54	M	65	Former (30 p/y)	Larynx	4a	0	0	wt	wt
57	M	61	Active (40 p/y)	Larynx	1	0	0	wt	wt
59	M	67	Former (40 p/y)	Larynx	3	0	0	wt	wt
71	M	64	Never	Larynx	1	0	0	wt	wt
81	M	73	Never	Larynx	2	0	0	C228T (0.11%)	wt
7	F	48	Active (42 p/y)	Larynx	3	0	0	wt	wt
10	M	67	Former (12 p/y)	Oropharynx	1	HPV16	HPV16	wt	wt
24	M	63	Former (12 p/y)	Oropharynx	1	HPV16	HPV16	wt	wt
32	M	71	Never	Oropharynx	2	HPV16	HPV16	wt	wt
33	M	45	Never	Oropharynx	4a	HPV16	HPV16	wt	wt
46	M	66	Never	Oropharynx	1	HPV16	HPV16	wt	wt
62	M	59	Never	Oropharynx	1	HPV16	HPV16	wt	wt
68	M	65	Never	Oropharynx	4b	0	0	wt	wt
74	M	76	Former (12 p/y)	Oropharynx	3	HPV16	HPV16	wt	wt
94	M	58	Never	Oropharynx	3	0	0	wt	wt
31	M	64	Active (51 p/y)	Oropharynx	4a	0	0	wt	wt
36	M	56	Active (20 p/y)	Oropharynx	2	HPV16	HPV16	wt	wt
8	M	52	Active (35 p/y)	Oropharynx + Hyphopharynx	2	0	0	wt	wt

*Pack/year, number of packs of cigarettes smoked per day by the number of years the person has smoked
**Galati et al. (2025)

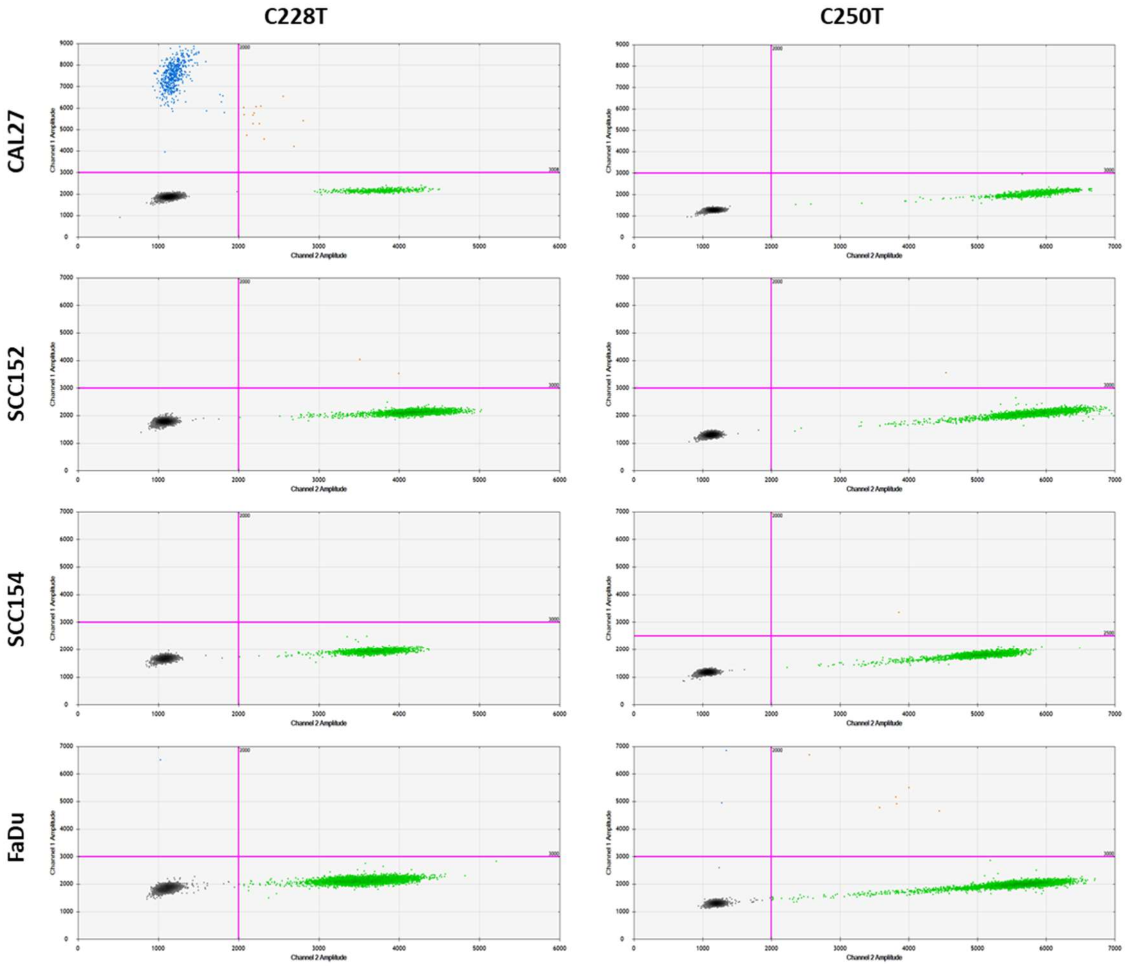

Figure 5S. Two-dimensional scatterplots of the droplet digital PCR TERTp C228T and C250T assays on CAL27, SCC152, SCC154 and FaDu cell lines.

Table 3S. Characteristics of HNSCC cell lines used in the study.

Cell lines	ATCC	Anatomical site	HPV DNA	TERTp mutation
CAL27	CRL-2095	Tongue	Neg	C228T
SCC152	CRL-3240	Tongue	HPV16	WT
SCC154	CRL-3241	Tongue	HPV16	WT
FaDu	HTB-43	Pharynx	Neg	WT
